# Semantic Mapping for Autonomous Subsea Intervention

**DOI:** 10.3390/s21206740

**Published:** 2021-10-11

**Authors:** Guillem Vallicrosa, Khadidja Himri, Pere Ridao, Nuno Gracias

**Affiliations:** Underwater Robotics Research Center (CIRS), Computer Vision and Robotics Institute (VICOROB), Universitat de Girona, Parc Científic i Tecnològic de la UdG. C/Pic de Peguera 13, 17003 Girona, Spain; khadidja.himri@udg.edu (K.H.); pere@eia.udg.edu (P.R.); ngracias@silver.udg.edu (N.G.)

**Keywords:** 3D object recognition, point clouds, global descriptors, semantic segmentation, semantic information, Bayesian probabilities, laser scanner, underwater environment, pipeline detection, inspection, maintenance and repair, AUV

## Abstract

This paper presents a method to build a semantic map to assist an underwater vehicle-manipulator system in performing intervention tasks autonomously in a submerged man-made pipe structure. The method is based on the integration of feature-based simultaneous localization and mapping (SLAM) and 3D object recognition using a database of a priori known objects. The robot uses Doppler velocity log (DVL), pressure, and attitude and heading reference system (AHRS) sensors for navigation and is equipped with a laser scanner providing non-coloured 3D point clouds of the inspected structure in real time. The object recognition module recognises the pipes and objects within the scan and passes them to the SLAM, which adds them to the map if not yet observed. Otherwise, it uses them to correct the map and the robot navigation if they were already mapped. The SLAM provides a consistent map and a drift-less navigation. Moreover, it provides a global identifier for every observed object instance and its pipe connectivity. This information is fed back to the object recognition module, where it is used to estimate the object classes using Bayesian techniques over the set of those object classes which are compatible in terms of pipe connectivity. This allows fusing of all the already available object observations to improve recognition. The outcome of the process is a semantic map made of pipes connected through valves, elbows and tees conforming to the real structure. Knowing the class and the position of objects will enable high-level manipulation commands in the near future.

## 1. Introduction

State-of-the-art autonomous underwater vehicles (AUVs) are commonly used for seafloor mapping in predominantly flat environments using multiple sensors, including side-scan sonar (SSS), multibeam echosounder (MBES), forward-looking sonar (FLS) and cameras, among others. The use of unmanned underwater vehicles (UUVs) for inspection, maintenance and repair (IMR) applications is nowadays limited to the use of remotely operated vehicles (ROVs) in inspection and/or intervention tasks. Nevertheless, during the last decade, the research community has made a significant effort defining a new class of UUV, the intervention autonomous underwater vehicle (I-AUV). This class of vehicles is expected to replace intervention ROVs in IMR tasks in the future [1]. Though several autonomous manipulation tasks have already been demonstrated, often only in water tank conditions, most are just proof of concept demonstrations oriented to very particular targets. Tasks such as valve turning [2,3], connector plug/unplug [4] and object search and recovery [5] are clear examples. Nevertheless, in all these tasks, custom algorithms have usually been used to detect and track a particular manipulation goal. Often the targets have been labeled with markers to simplify the problem, or the robot was limited to performing a particular manipulation action over a particular target object. In contrast, a truly autonomous I-AUV should be able to obtain and use semantic knowledge of its surroundings. As such, the vehicle should be capable of identifying which objects are around it, which class they belong to, and which tasks can be performed on them. For instance, if a safety valve has to be manipulated in case of an alarm, the I-AUV needs to know which valve, where it is and how it can be opened or closed. This leads to the semantic map concept—a map containing the objects position and their specific class. Semantic mapping is a key technique to endow the I-AUV with autonomous reasoning capabilities.

### 1.1. Objectives

This paper tackles the semantic map building problem for an I-AUV equipped with a real-time high-resolution laser scanner and working on IMR operations. It extends our prior work [6], where a point feature-based 3D object recognition method was proposed. The method used Bayesian estimation as a probabilistic framework to integrate multiple detections into a single, and more robust, object class identification. To do so, it was necessary to track objects along the sequentially grabbed scans. For this purpose, a Interdistance Joint Compatibility Branch and Bound (IJCBB) object tracking method was proposed, which was able to track the objects in the presence of navigation glitches due to sporadic failures of the the Doppler velocity log (DVL) measurements. Moreover, the method exploited semantic information related to object pipe connectivity (number of pipes connected to the object) to constrain the potential set of compatible object classes used during the Bayesian estimation. Nevertheless, the IJCBB must establish at least three pairings between two scans to be able to register them. Otherwise, the tracking fails and the object detections in this scan cannot contribute to the Bayesian estimation. On the other hand, the iterative nature of the tracking algorithm reduces the drift, but is not able to cancel it. Therefore, the natural next step is to employ simultaneous localization and mapping (SLAM) techniques using the pipes and objects as features to build a drift-less consistent map of the structure. Using conventional data association algorithms, between the objects in a scan and the objects in the SLAM, it is possible to track the objects and apply the Bayesian estimation. The outcome of the process is a semantic map of pipes and objects, which provides the I-AUV with an accurate navigation as well as with the semantic knowledge of the manipulable objects around it.

### 1.2. Contributions

The main contributions of the present paper are the following:

- A feature-based extended Kalman filter (EKF) SLAM method is proposed which uses line and point features to represent the pipes and the objects, respectively. The method solves two problems: (1) it provides a drift-less navigation; and (2) it assigns a globally consistent identifier to every object in every scan, enabling Bayesian estimation. When conveniently combined with the object recognition results, it becomes a semantic map endowing the I-AUV with the semantic knowledge required to perform high-level commands, such as *Open Valve X*, for example.
- It provides a method for plane segmentation which partitions the point cloud according to the average maximum curvature and classifies the partitions either as planes or as a curved region. The method allows separation of the flat surfaces corresponding to the walls of the water tank, where the experiment was performed, from the pipe structure itself.
- It provides an extension to the semantic object segmentation method already proposed in [6], ensuring the correct segmentation of the valve handle, which proved problematic in the previous paper.

### 1.3. Structure of the Paper

The remainder of the paper is organized as follows. Section 2 describes the state of the art on underwater SLAM, object recognition and semantic mapping. Section 3 describes the object recognition pipeline from the segmentation of the scans to the Bayesian recognition. Section 4 describes the feature-based SLAM for object and pipe feature tracking. Section 5 describes the experimental setup and the results obtained. Section 6 and Section 7 provide conclusions and future work on the results obtained.

## 2. State of the Art

### 2.1. Underwater SLAM

Many outdoor field robots rely on absolute measurements to bound the dead reckoning (DR) navigation drift, such as the Global Positioning System (GPS). However, in underwater robotics, those sensors are unavailable due to electromagnetic attenuation; underwater robots instead have to rely on acoustic localization methods such as long baseline (LBL) [7], short baseline (SBL) [8], ultra-short baseline (USBL) [9] or GPS intelligent buoys (GIB) [10]. Those methods require deployment of the beacons and/or a support vessel to provide the gps positioning to be composed with the measured acoustic position. Unfortunately, those methods restrict the vehicle to a predefined zone (lbl) or decrease their precision with increasing depth of the vehicle (SBL, USBL and GIB).

A solution to overcome these issues and have a completely independent AUV is to correlate the vehicle sensor measurements with a map of the environment to reliably locate its position with Terrain-Based Navigation (TBN) techniques [11]. However, precise maps are not widely available, and so many researchers rely on SLAM methods, where the robot incrementally builds a model of the environment and simultaneously uses it to estimate its position within it.

Underwater SLAM can be categorised according to the type of sensors used to perceive the environment. On the one hand, vision-based sensors perceive the environment at high rates and high precision, but they are very sensitive to water visibility, which greatly limits their range. On the other hand, acoustic-based sensors provide low-rate and low-precision measurements regardless of visibility. Regarding acoustic SLAM, we can further classify SLAM into feature-based and featureless methods. Feature-based methods are generally used in man-made environments, where features are easier to extract [12,13,14], while featureless methods are primarily used in natural environments [15,16,17,18,19,20,21].

In contrast, underwater vision-based SLAM relies heavily on visual features extracted from the texture of the environment [22,23,24,25,26,27]. If the environment is texture-less, an alternative is to use laser-camera systems, where the laser produces the necessary texture to extract point clouds from the environment. Initial developments of this approach relied on a fixed laser scanner that, combined with the vehicle motion, produces the point clouds [28,29], but suffers from navigation drift.

A new laser scanner based on a moving mirror provides scans at a maximum rate of 6 Hz, fast enough to allow the vehicle drift during a single scan to be neglected [30]. This laser scanner has already been tested on motion planning applications in an unknown environment [31] and in a pose-based SLAM for mapping [32]. In the present work, we focus on the application of this laser scanner to semantically extract features that serve as input for the SLAM algorithm and ease the recognition of the object features on pre-trained models of the different objects.

### 2.2. Object Recognition

Object recognition is a domain of 3D scene exploration and understanding associated with applications such as autonomous driving and housekeeping robots. 3D object recognition has emerged thanks to pre-existing 2D methods translated into 3D and the advanced availability of different types of 3D sensors.

In the field of object recognition based on point clouds, several surveys have been carried out in which methods and ideas based on global and local descriptors have been presented [33,34,35]. Global recognition methods interpret the entire object as a unique vector of values, while local recognition methods focus more on a local region and are computed from salient points. Recently, deep learning has gained increasing attention. The following two publications are representative examples. In [36], Guo et al. summarized deep learning methods applied to 3D point clouds. The authors aimed to select the most relevant applications for point cloud understanding, considering 3D shape classification, 3D object detection and tracking, and 3D point cloud segmentation. They evaluated the quality of the performance of state-of-the-art methods based on deep learning and compared the methods with different publicly available datasets. In Tian et al. [37], the authors proposed a dynamic graph convolutional broad network (DGCB-Net) for feature extraction and object recognition from point clouds, and their method was tested on several public datasets and one dataset which they collected.

However, fewer papers have focused on underwater application scenarios, with the exception of the paper by Martin et al. [38], in which a processing pipeline is presented, based on the use of a deep PointNet neural network. The proposed method was able to detect pipes and valves from 3D RGB point clouds in underwater environments using a generated dataset to train and test the network. Recent work by Pereira et al. [39] is also based on a deep learning approach, where a convolutional neural network was used for recognizing a docking structure from point clouds. Their methods were evaluated with simulated and real datasets.

Although deep learning approaches have been reported to have attained accurate results, such methods are very demanding in terms of the amount of training data to ensure proper learning generalization. In the case of man-made structures observed by sensors that provide only colourless point clouds, the collection of the required training data is a difficult and time-consuming task.

The work described in Martin et al. [38] used a similar man-made structure as the one in our work, comprising valves interconnected by pipes. Furthermore, the experiments in both papers were conducted in an underwater environment. Their work is directly related to the problem we are trying to solve, i.e., the recognition of man-made objects underwater, because it formed part of the same research project TWINBOT [40] in which both groups participated. In the following paragraphs, we provide a comparison of the two works, which highlights the trade-offs between the two approaches.

- In the present work, we have used a feature-based SLAM approach to object recognition using a 3D point cloud with no RGB information, obtained with a laser scanner. The process can be summarised as follows:
  - The segmentation of the ground was performed using the methods explained in Section 3.2. The segmentation of pipes was performed separately from the recognition of the objects;
  Five object classes were defined in the experiments, which were segmented based on the pipe connections;
  - The knowledge database was generated from the object’s CAD model using a process described in our previous article [33]. The test data was collected in the test pool of our laboratory, and included 1268 point clouds for individual objects, extracted from 245 laser scans;
  - The main recognition performance results are found in Section 5.4.
- In the work of Martin et al. [38], a deep learning approach was applied for the detection of pipes and valves. The network used, as input, 3D point clouds with RGB information obtained from stereo cameras, and the following steps were performed:
  - Ground truth data were manually created from the point cloud, and divided into three classes: pipes, valves and background;
  - Two datasets were used. The first dataset was acquired in a test tank and contained 262 point clouds. This dataset was divided into two subsets, the first containing 236 point clouds which were used to train the network and the remainder used as test samples. The second dataset was collected in the sea and included 22 point clouds that were used only as a test set;
  - 13 experiments were conducted varying the hyper-parameters in the training phase: batch size, learning rate, block-stride and number of points;
  - To assess the performance of the neural network and estimate how the model is expected to perform, a 10-fold cross-validation was performed. Overall, 9 subsets of 213 point clouds were used for training, and 1 subset of 23 point clouds was used for testing. The final classification result was obtained by averaging the performance of these ten different results;
  - From the results presented, it can be seen that the background class was predominant, followed by the pipe and valve classes in both pool and sea experiments.

### 2.3. Semantic Mapping

Semantic mapping started indoors with scene recognition [41,42,43,44] and then moved outdoors. It has been applied on various input data, such as cameras [45,46], depth cameras [47,48] or laser scanners (usually LIDARs) [49,50,51]. Implementations vary from surpervised to unsupervised methods, where semantic classes are a priori unknown.

Adding semantic information to underwater maps contributes to a better spatial awareness of nearby terrains and objects, enabling higher-level tasks to be performed. This is especially important for imr tasks where robots have to be aware of the different components and how to interact with them.

In the underwater environment, it has been mainly used for semantic image segmentation [52,53], which can also be applied to exploration [54]. To the best of the authors’ knowledge, semantic mapping has not yet been applied to point clouds obtained underwater with a laser scanner for imr tasks, and thus, this paper goes beyond the state of the art.

## 3. Object Recognition Pipeline

**Figure 1:**
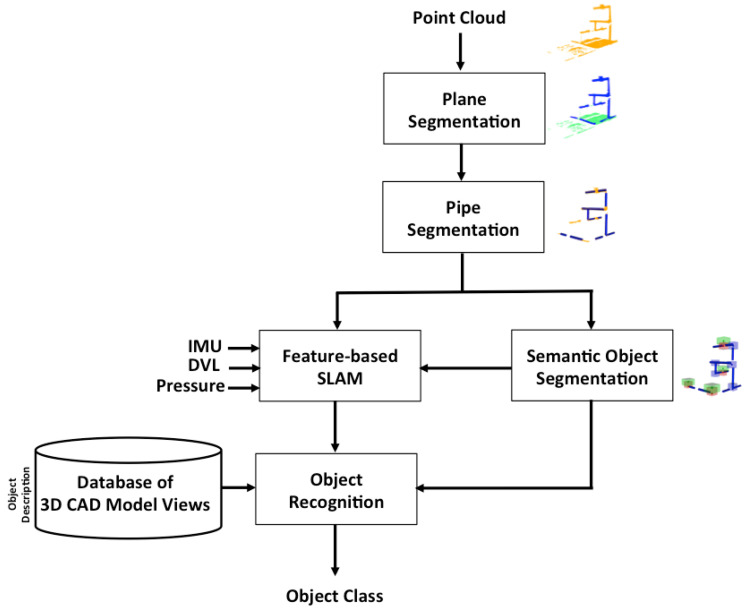
3D object recognition pipeline.

As can be seen in Figure 1, the object recognition pipeline is divided into several modules. First, the floor and lateral walls/slopes of the water tank where the experiment takes place are segmented and subtracted from the scanned point-cloud. Then, pipes are detected and the resulting point cloud is used as input for the semantic object segmentation. Having extracted the planes and the pipes from the scan, objects are segmented.

A feature-based SLAM is continuously running, integrating DVL, pressure and heading reference system (AHRS) measurements. The input pipes and objects are used as features of the SLAM, which simultaneously estimates the robot pose, and the position of the already-observed pipes and objects. Therefore, solving the association of the objects segmented from the scan with those already mapped, it is possible to associate a global identifier with them. Finally, the object recognition module uses the point feature descriptors of the partial views of the segmented objects, matching them against those stored in the object database, identifying the object class. Since the global identifier of the observed object instance is known thanks to the SLAM output, it is possible to use several past object class estimations to compute its global object class, achieving more robust results. Hereafter, the different modules are described in more detail.

### 3.1. Object Data Base

A database of point clouds was created (Table 1), containing overlapping partial views of isolated objects. These views were created from 3D CAD models and captured using a virtual camera. This database was useful for the design of simulated experiments and for their statistical analysis, as presented in our previous work [6]. Details on the creation of the database can be found in the same publication.

### 3.2. Plane Segmentation

In our previous work [6], planes were detected using random sample consensus (RANSAC). Unfortunately, in several scans, the principal plane detected did not correspond to the floor or the walls of the water tank. Sometimes, points belonging to different pipes and even objects, and others belonging to the slopes, became co-planar, forming the most significant plane in the scene. However, removing it would wrongly eliminate a significant number of points in the pipes and objects, making the recognition more challenging. To avoid this problem, an alternative method is proposed in this paper.

The problem of plane segmentation can be seen as an unsupervised classification problem, where the goal is to group the points into regions defined according to their curvature, which is an attribute describing the local geometry around a point. In Point Cloud Library (PCL), the curvature of a point is computed performing an eigen-decomposition of the points in the neighbourhood. The eigenvector corresponding to the smallest eigenvalue provides the direction of the normal, and the other two provide the tangent plane. The curvature κ is defined as the ratio between the smallest eigenvalue and the addition of the three eigenvalues:

(1) $$\kappa =\frac{\lambda_{0}}{\lambda_{0}+\lambda_{1}+\lambda_{2}}\;\mathrm{where}\;\lambda_{0}<\lambda_{1}<\lambda_{2}.$$

**Figure 2:**
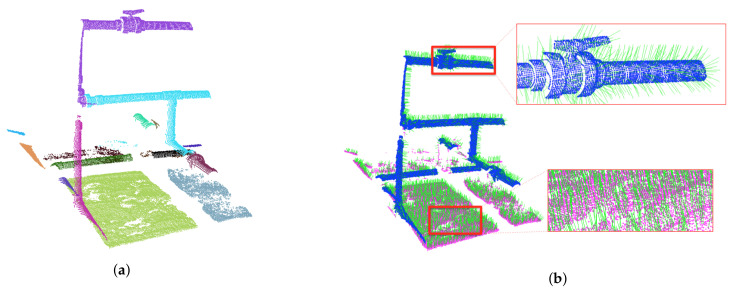
Plane segmentation. (**a**) Outcome regions of the region-growing method. (**b**) Segmentation of the point cloud into two regions with normals in green: (I) non-flat areas in blue, and (II) flat areas in pink.

To remove the planar surfaces, first we segmented the point cloud into several regions using the region-growing method [55]. The algorithm begins by selecting as a seed point the one with least curvature. Then, the region is computed by growing the seed to those adjacent points in the neighbourhood whose angles between normals (the normal of the seed and the local normal at the point) are within a pre-defined threshold. Next, the points within the region with a curvature below a threshold are considered as new seeds, and the algorithm is iterated until no more seeds are available. At this point, the first region has been segmented and the algorithm is applied again to the rest of the point cloud. The result is a set of regions having a smooth evolution of the angle among their normals. The regions are separated either for having a sudden change in their normals (smoothness), or because they are spatially separated, as shown in Figure 2. The threshold angle between the normal vectors was set to 30 degrees. If the points are on the same plane, then the normals of the fitting planes of these two points are approximately parallel.

**Figure 3:**
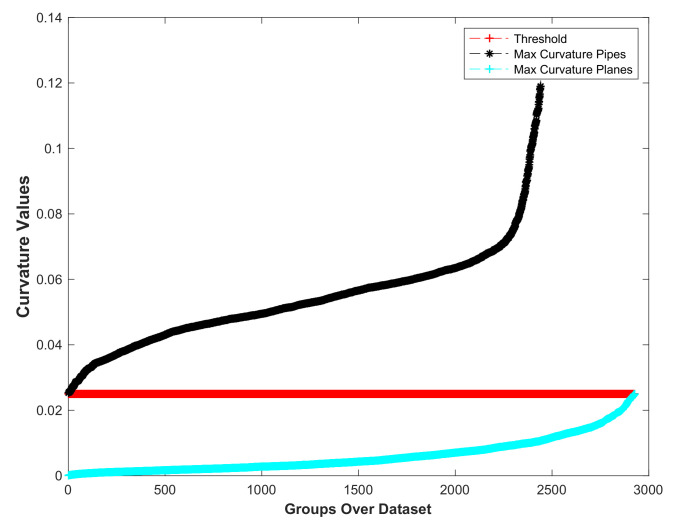
Mean curvature threshold separating the pipes from the flat areas. The horizontal axis represents, for all the dataset scans, the regions obtained using the region-growing method. The vertical axis provides, for each region, its mean curvature.

Second, the resulting regions are classified into two categories based on an empirical threshold on their mean curvature (Figure 2). We evaluated the curvature of each region in a neighbourhood of 50 points and chose an empirical threshold of 0.025 (Figure 3). Each region from the growing regions result is classified as: (a) points on flat areas such as the bottom and the slopes on both sides of the water tank, or (b) points on the rest of the cloud, such as objects and pipes of the structure.

Subsequently, the flat regions are deleted, and the remainder are merged into a single region containing the non-flat areas to be further processed. The proposed plane segmentation method is shown in Algorithm 1.
**Algorithm 1:** Plane Segmentation
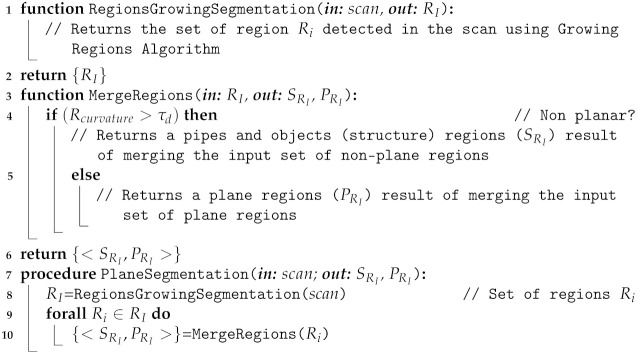


### 3.3. Pipe Detection

For detecting pipes in the current scan, a method based on the RANSAC implementation in PCL was used. This method models the pipes as cylinders with seven parameters, consisting of the 3D position of a point on the axis, axis direction, and cylinder radius. The scan is divided into two categories, namely the pipe cloud category and non-pipe cloud category. Since the radius of the pipes is known and objects have a maximum size, only the segmented cylinders with length more than 0.30 m and maximum radius of 0.064 m are considered as pipes. To calculate the endpoints of the pipes, the selected set of points is projected onto the pipe axis, and the points at the extreme ends are considered as the limits of the pipe.

**Figure 4:**
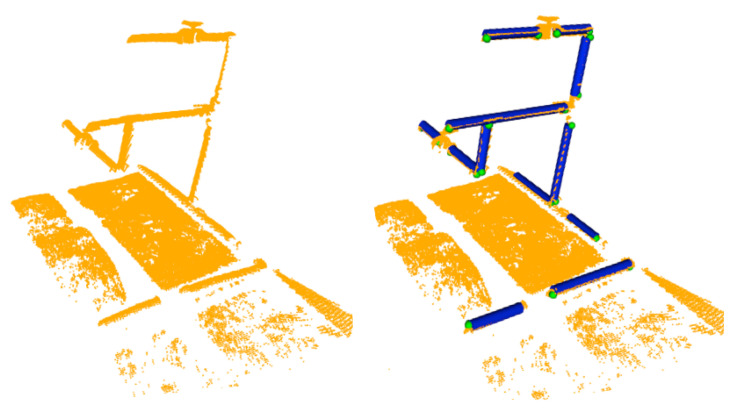
Pipe detection: (**left**) 3D laser scan point cloud; (**right**) Pipes in blue with their respective endpoints in green.

Scan deformations caused by motion-induced distortions during the acquisition of the laser scan [32] can occasionally lead to two different detections being generated for the same pipe. The solution for such cases as well as details on the implementation of the pipe detection are provided in [6]. An example of pipe detection with their respective endpoints is given in Figure 4.

### 3.4. Semantic Object Segmentation

**Figure 5:**
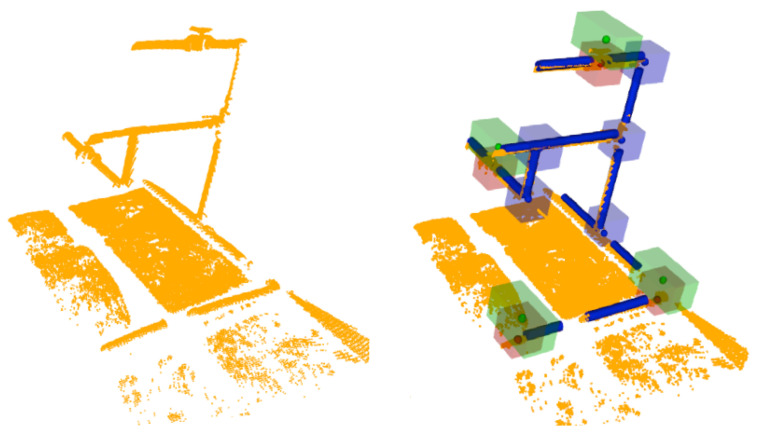
Semantic object segmentation: (**left**) 3D laser scan point cloud; (**right**) Example of segmentation and how objects with different connectivity are treated differently.

The proposed semantic 3D object segmentation is inspired and motivated by the fact that objects are found at the extremities of pipes. Knowledge about these objects includes detailed information about the connectivity of the objects and structural knowledge, such as the fact that valves with two parallel connections are characterised by handles, which is an important feature for objects like butterfly valves to distinguish them from their homologous valves. In addition, functional knowledge is needed for these features, allowing the robot to infer whether the valves can be turned on or off based on the position of a handle. To this end, the semantic segmentation problem can be formulated as follows: Given an object with one or two parallel connections (Figure 5), it is possible to find a potential handle, as shown in the right part of the figure, where objects with one or two parallel connections are segmented using a ’mushroom’ shape (green cube on the top of the red one). The base is defined for the body of the object and the parallel pipe shape for the potential handle, while if the object has more connections or two perpendicular connections, only the base is segmented, as shown with blue cubes.

### 3.5. 3D Object Recognition Based on Global Descriptors

Object recognition is an essential part of building a semantic map of the environment. In [33] we studied and compared several descriptors using synthetic and real data. The best results involving experimental data were achieved using the Clustered Viewpoint Feature Histogram (CVFH) [56] descriptor, which is therefore used in this paper.

### 3.6. Bayesian Recognition

A disadvantage of object recognition with a single-view approach is that multiple objects may have similar views. A study based on the confusion matrices for the various objects was carried out in our previous work [33]. Given a set of observations of a particular object, we can use confusion matrices to determine how many observations were recognized as *object-class-n*, where *n* indicates the class name of the object. Given this information, we can estimate a probability for each class as well as the confusion between classes, which is used to implement a Bayesian estimation method to improve object recognition results.

For this purpose, several observations were combined to calculate the probability that an object belongs to each object class. The selected object was assigned to the class with the highest probability. This method required continuous observation of the same objects across the scans, so a tracking method was required to iteratively compute the Bayesian probabilities. In our previous work [6], this tracking was performed using a navigation-less variant of the Joint Compatibility Branch and Bound (JCBB) algorithm, based on the distances between objects within a scan, and referred to as IJCBB. In the present work, we use the SLAM solution described in Section 4, which achieves significantly higher performance.

### 3.7. Bayesian Estimation

In order to solve the common problem of ambiguous observations caused by having only partial views of the objects in the scans, a Bayesian estimator is applied. In [33] we have already computed the object confusion matrix; this matrix is used as an estimate of the required conditional probabilities. The object class recognised with the global descriptor is denoted as $Z_{C}$. *X* is the actual class of this object, and *Ball-Valve*, *Elbow*, *R-Tee*, *R-Socket*, *Butterfly-Valve*, *3-Way-Valve* are potential class candidates, sub-indexed with numbers 1 to 6 respectively. $P(Z_{C}|X_{i})$ indicates the probability that the object is recognised as class $Z_{C}$ when its actual class is $X_{i}$. If C=i then it is a true positive (TP), otherwise (C≠i) it is a false positive (FP).

### 3.8. Semantic-Based Recognition

By knowing the number of pipes connected to the object and their geometry, the recognition rate can be further improved. This method was presented in [6] and is briefly summarized here for completion.

The information about the number of pipe connections and their geometry is used to reduce the set of possible classes for a given object by considering only those classes that are compatible with that configuration. For example, if we know that an object is connected to 3 pipes, then only 2 candidate classes are possible: the *R-Tee* and the *3-Way-Valve*. Thus, the Bayesian probabilities are computed only for the compatible candidate classes and considered zero for the rest.

Four different geometric configurations may arise:

**Configuration 1** Three pipes: two collinear and one orthogonal. This group contains the *R-Tee* and the *3-Way-Valve*;
**Configuration 2** Two orthogonal pipes: This group contains the *Elbow* but also the members of the previous group, since it is possible that the third pipe has not yet been observed;
**Configuration 3** Two collinear pipes: All objects are included in this group, except the *Elbow* and the R-Sockets. The remaining objects admit a collinear connection to two pipes;
**Configuration 4** Single or no connection: All objects are considered as potential candidates.

It can be seen from Table 2 that these configurations have a hierarchy in the sense that the first is the most restrictive, the second is less restrictive and encompasses the objects of the first group, and so on. One exception is group 3, for 2 collinear pipes where the *Elbow* of group 2 is not present. It is worth noting that the laser scanning process often provides only partial views of the objects due to occlusions and the limited field of view. As such, a certain object may appear as connected to a single pipe in the first observation, then connected to three pipes on the second observation and then just to a single pipe in the third observation. Since objects are mapped in the SLAM, we can use the knowledge of the previously observed configurations to better compute the probabilities. As an example, if an object is observed in configuration 1 and then configuration 2, then the probabilities for the second observation will be computed as for configuration 1 (which is the most restrictive).

## 4. Simultaneous Localization and Mapping for Object and Pipe Tracking

Once the pipes and objects are segmented from the scans, they are sent as input to a SLAM algorithm that integrates AUV navigation with those features in order to improve navigation and track the features, keeping a single global ID for each of them. The output of the SLAM to the semantic Bayesian recognition are the global IDs for each object detected in the scan. This ensures that different observations of the same object are used together to better estimate the object class.

### 4.1. Line Feature Representation

**Figure 6:**
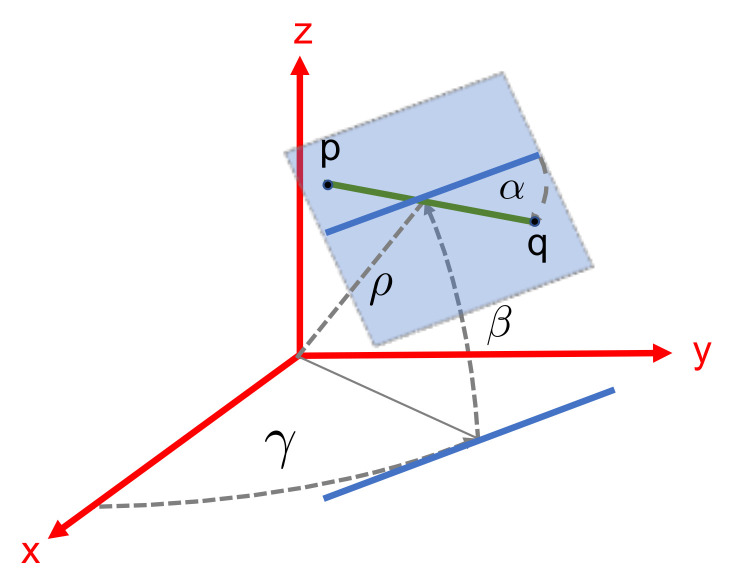
Line feature parametrization.

The pipes are represented using an ortho-normal line representation [57] consisting of three angles of rotation (αβγ) and the shortest distance from the frame origin to the line ρ (2) (Figure 6).

(2) $$L=\left[\begin{array}{cccc} \alpha & \beta & \gamma & \rho \end{array}\right]$$

**Figure 7:**
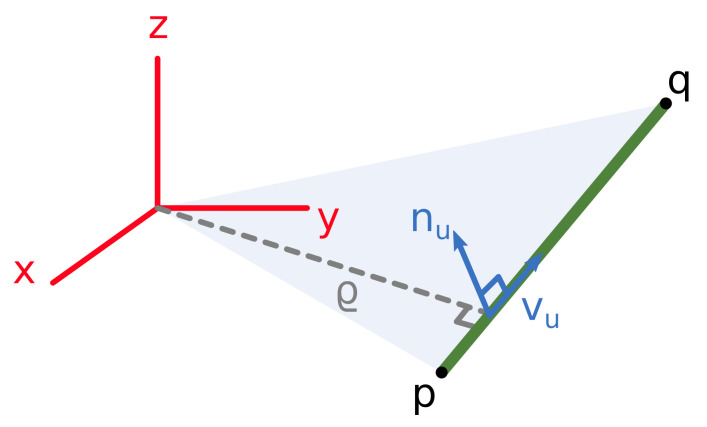
Ortho-normal representation of a pipe segment.

Given the segment endpoints (p and q) provided by the pipe detection algorithm (see Section 3.3), the ortho-normal representation is computed using Plücker coordinates [58].

(3) $$\begin{array}{cc} n & =p\times q \end{array}$$

(4) $$\begin{array}{cc} v & =q-p \end{array}$$

(5) $$\begin{array}{cc} n_{u} & =n/||n|| \end{array}$$

(6) $$\begin{array}{cc} v_{u} & =v/||v|| \end{array}$$

(7) $$\begin{array}{cc} r_{u} & =v_{u}\times n_{u} \end{array}$$

(8) $$\begin{array}{cc} R & =\left[\begin{array}{ccc} r_{u} & v_{u} & n_{u} \end{array}\right]=Rot\left(\gamma ,z\right)Rot\left(\beta ,y\right)Rot\left(\alpha ,x\right) \end{array}$$

(9) $$\begin{array}{cc} \rho & =||n||/||v|| \end{array}$$

where $v_{u}$ represents the line direction and $n_{u}$ is perpendicular to the plane formed by the two endpoints and the frame origin (Figure 7). The three angles of rotation can be extracted from the rotation matrix R as:

(10) $$\begin{array}{cc} \alpha & =atan2(v_{u_{z}},n_{u_{z}}) \end{array}$$

(11) $$\begin{array}{cc} \beta & =\mathrm{asin}(r_{u_{z}}) \end{array}$$

(12) $$\begin{array}{cc} \rho & =atan2(r_{u_{y}},r_{u_{x}}) \end{array}$$

The line is computed from the pipe endpoints which are known in the vehicle sensor frame {S}, which is the frame of reference of the point cloud. Therefore it is initially referenced to {S} and has to be transformed to the world frame {W}:

(13) $$\begin{array}{cc} {}^{W}\rho \cdot {}^{W}n_{u} & ={}^{W}R_{S}\cdot {}^{S}\rho \cdot {}^{S}n_{u}+{}^{W}t_{S}\times \left({}^{W}R_{S}\cdot {}^{S}v_{u}\right) \end{array}$$

(14) $$\begin{array}{cc} {}^{W}v_{u} & ={}^{W}R_{S}\cdot {}^{S}v_{u} \end{array}$$

where ${}^{W}r_{S}$ and ${}^{W}t_{S}$ are, respectively, the rotation and the translation that transform from the sensor frame {S} to the world frame {W}.

Similarly, the opposite transformation is computed as:

(15) $$\begin{array}{cc} {}^{S}\rho \cdot {}^{S}n_{u} & ={}^{W}R_{S}^{T}\cdot {}^{W}\rho \cdot {}^{W}n_{u}-{}^{W}R_{S}^{T}\cdot {}^{W}t_{S}\times {}^{W}v_{u} \end{array}$$

(16) $$\begin{array}{cc} {}^{S}v_{u} & ={}^{W}R_{S}^{T}\cdot {}^{W}v_{u} \end{array}$$

From this, we can calculate the frame change Jacobians with respect to the line representation in the frame and to the sensor position in the world.

### 4.2. State Vector

The state is represented with the Gaussian random vector $x\left(k\right)$:

(17) $$x\left(k\right)={\left[\begin{array}{ccccc} x_{v}\left(k\right) & f_{1}\left(k\right) & f_{2}\left(k\right) & \cdots & f_{n}\left(k\right) \end{array}\right]}^{T}$$

defined by 2 parameters, the mean:

(18) $$\hat{x}\left(k\right)={\left[\begin{array}{ccccc} {\hat{x}}_{v}\left(k\right) & {\hat{f}}_{1}\left(k\right) & {\hat{f}}_{2}\left(k\right) & \cdots & {\hat{f}}_{n}\left(k\right) \end{array}\right]}^{T}$$

and the covariance matrix p(k), which provides the covariance of the vehicle and the feature lines, as well as their cross-correlations:

(19) $$p\left(k\right)=E\left(\left[x\left(k\right)-\hat{x}\left(k\right)\right]{\left[x\left(k\right)-\hat{x}\left(k\right)\right]}^{T}\right)=\left[\begin{array}{cccc} p_{v}\left(k\right) & p_{{vf}_{1}}\left(k\right) & \cdots & p_{{vf}_{n}}\left(k\right)\\ p_{f_{1}v}\left(k\right) & p_{f_{1}}\left(k\right) & \cdots & p_{f_{1}f_{n}}\left(k\right)\\ \vdots & \vdots & \ddots & \vdots \\ p_{f_{n}v}\left(k\right) & p_{f_{n}f_{1}}\left(k\right) & \cdots & p_{f_{n}}\left(k\right) \end{array}\right]$$

The vehicle state $x_{v}={\left[x\;y\;z\;\phi \;\theta \;\psi \;u\;v\;w\right]}^{T}$ has nine dimensions, including vehicle position ${\left[x\;y\;z\right]}^{T}$ and the vehicle orientation $\left[\phi \;\theta \;\psi \right]$, both represented in the world reference frame {W}. This frame is located at the water surface, being aligned with the north (i.e., north-east-down (NED) reference frame). The linear velocities ${\left[u\;v\;w\right]}^{T}$, instead, are referenced to the vehicle’s frame {B}. This is also the minimum dimension of the state vector at the beginning of the execution. The state vector is initialized with the vehicle at rest on the surface when the first depth, ahrs and DVL measurements are received.

The line and object features $\left[f_{1}\left(k\right)\;f_{2}\left(k\right)\;\cdots \;f_{n}\left(k\right)\right]$ are static and defined in the world reference frame {W}. Line features are represented with ortho-normal coordinates (see Section 4.1) and objects are represented by their coordinates xyz. The number of line features in the state vector is represented by $n_{l}$ and the number of object features is represented by $n_{o}$, with the total number of features being $n=n_{l}+n_{o}$.

### 4.3. Prediction

A six degrees of freedom (DoF) constant-velocity kinematics model is used to predict the vehicle state evolution from time k−1 to time *k*. The attitude rate of change (Euler angle derivatives), available from the AHRS, is used as the system input ($u\left(k\right)={\left[\dot{\phi }\;\dot{\theta }\;\dot{\psi }\;\right]}^{T}$). The uncertainty is modeled as a white Gaussian noise in linear acceleration ($w_{l}$) and attitude velocity ($w_{a}$). This model can be formulated as:

(20) $$\begin{array}{cc} x_{v}\left(k|k-1\right) & =f\left(x_{v}\left(k-1\right),u\left(k\right),w\left(k\right)\right) \end{array}$$

(21) $$\begin{array}{cc} x_{v}\left(k|k-1\right) & =\left[\begin{array}{c} \left[\begin{array}{c} x\\ y\\ z \end{array}\right]+Rot\left(\phi ,\theta ,\psi \right)\left(\left[\begin{array}{c} u\\ v\\ w \end{array}\right]\Delta t+w_{l}\frac{\Delta t^{2}}{2}\right)\\ \left[\begin{array}{c} \phi \\ \theta \\ \psi \end{array}\right]+\left(u+w_{a}\right)\Delta t\\ \left[\begin{array}{c} u\\ v\\ w \end{array}\right]+w_{l}\Delta t \end{array}\right] \end{array}$$

where Δt is the time between k−1 and *k*, and $w=\left[w_{l}\;w_{a}\right]∼N\left(0,q\right)$ is a white Gaussian noise representing the uncertainty of the linear acceleration $w_{l}=\left[w_{\dot{u}}\;w_{\dot{v}}\;w_{\dot{w}}\right]$ and the attitude velocity $w_{a}=\left[w_{\dot{\phi }}\;w_{\dot{\theta }}\;w_{\dot{\psi }}\right]$. In contrast, the features are static and are kept constant throughout the prediction. Hence, the whole state can be predicted using:

(22) $$x\left(k|k-1\right)={\left[\begin{array}{ccccc} f\left(x_{v}\left(k-1\right),u\left(k\right),w\left(k\right)\right) & f_{1}\left(k-1\right) & f_{2}\left(k-1\right) & \cdots & f_{n}\left(k-1\right) \end{array}\right]}^{T}$$

### 4.4. Navigation Sensor Updates

The different navigation sensors present on the vehicle (pressure sensor, DVL and AHRS) provide direct observations of the state vector. Therefore, a linear observation model can be used. The general model in this case is:

(23) z(k)=H(k)·x(k|k−1)+m(k)

where z is the measurement vector, and $m\equiv N\left(0,R\right)$ is a white Gaussian noise vector with 0 mean and covariance R. The size of the observation matrix H, as well as the size of R, changes between the different types of observations.

A pressure sensor produces a 1 dof position measurement which is a direct observation of the vehicle’s depth (i.e., *z* position). Therefore, the resulting observation matrix is:

(24) $$H_{\mathrm{DEPTH}}\left(k\right)=\left[\begin{array}{ccccc} 0 & 0 & 1 & 0_{1\times 6} & 0_{1\times (4n_{l}+3n_{o})} \end{array}\right]$$

and $R_{\mathrm{DEPTH}}$ is the covariance of the pressure sensor:

(25) $$R_{\mathrm{DEPTH}}=\sigma_{\mathrm{DEPTH}}^{2}$$

An ahrs produces 3 dof angular measurements, which are direct observations of the vehicle attitude (Euler angles). The resulting observation matrix is:

(26) $$H_{\mathrm{AHRS}}\left(k\right)=\left[\begin{array}{cccc} 0_{3\times 3} & I_{3\times 3} & 0_{3\times 3} & 0_{3\times (4n_{l}+3n_{o})} \end{array}\right]$$

and the covariance matrix $R_{\mathrm{AHRS}}$ is a 3×3 square matrix with the uncertainties of each angle observation:

(27) $$r_{\mathrm{AHRS}}\left(k\right)=\left[\begin{array}{ccc} \sigma_{\phi }^{2} & 0 & 0\\ 0 & \sigma_{\theta }^{2} & 0\\ 0 & 0 & \sigma_{\psi }^{2} \end{array}\right]$$

A DVL produces 3 DoF velocity measurements, which are direct observations of the vehicle velocity in its own frame:

(28) $$H_{\mathrm{DVL}}\left(k\right)=\left[\begin{array}{cccc} 0_{3\times 3} & 0_{3\times 3} & I_{3\times 3} & 0_{3\times (4n_{l}+3n_{o})} \end{array}\right]$$

and the covariance matrix $R_{\mathrm{DVL}}$ is a 3×3 square matrix with the uncertainties of each velocity estimation.

(29) $$r_{\mathrm{DVL}}\left(k\right)=\left[\begin{array}{ccc} \sigma_{u}^{2} & 0 & 0\\ 0 & \sigma_{v}^{2} & 0\\ 0 & 0 & \sigma_{w}^{2} \end{array}\right]$$

### 4.5. Line Feature Observation

**Figure 8:**
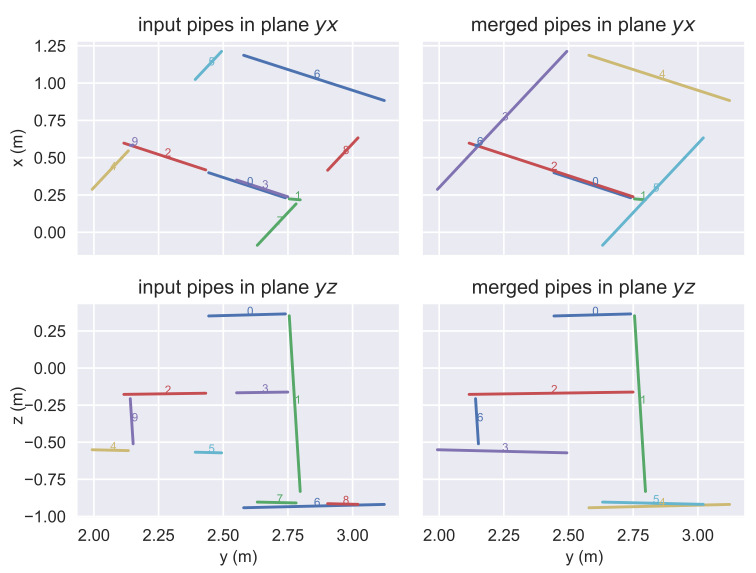
(**left**) Original pipes received from the pipe detector. (**right**) Merged pipes before SLAM update.

From the pipe detector (see Section 3.3), line features are received as pairs of endpoints in the sensor frame {S}. A first merging filter is used to join collinear segments onto bigger segments. This is done by checking the point-to-line distance of the endpoints against the line defined by the other segment and vice-versa. If all the distances are below a threshold, the segments are joined and the longest possible segment from the two pairs of endpoints is retained (Figure 8).

As observations of a highly angular structure, the angular threshold between lines is not very sensitive, and in this case, a maximum value of 0.175 rad is used. However, the distance threshold is more sensitive due to the existence of parallel lines. A maximum value around the half distance between the closest lines in the real structure, 0.3 m, is used.

The merged segments are converted to the line feature representation in the sensor frame using Equations (3)–(12). The first step in the feature update process is feature association. Already mapped features in the state vector are transformed to the sensor frame together with their uncertainty. A JCBB algorithm is used to ensure consistency in the associations, as opposed to standard individual compatibility [59]. Once this association is solved, we have two kinds of observations: re-observed features that were already in the state vector, or new features that are candidates to be added to the state vector.

For better representation of the line features when observing the results, the endpoints provided by the pipe detector are saved and re-projected to their associated line at the end of every feature observation.

#### 4.5.1. Line Feature Re-Observation

Given a feature observation z(k), associated with an already mapped feature $f_{j}$, the non-linear observation equation is defined as:

(30) $$z\left(k\right)=h_{f_{j}}\left(x\left(k\right),v_{j}\left(k\right)\right)=h_{j}\left(x_{v}\left(k\right),f_{j}\left(k\right),v_{j}\left(k\right)\right)$$

(31) $$v_{j}\left(k\right)\equiv N\left(0,R_{f_{j}}\left(k\right)\right)$$

where the $h_{j}$ function uses the the robot pose $x_{v}$ and the feature parameters $f_{j}=\left[{}^{W}\alpha \;{}^{W}\beta {}^{W}\gamma \;{}^{W}\rho \right]$ are represented in the world frame to transform the line parameters to be referenced to the sensor frame. To do so, first, (3)–(7) are used to compute the vectors ${}^{W}r_{u}$, ${}^{W}v_{u}$, ${}^{W}n_{u}$ and ${}^{W}\rho$. Next, Equations (15)–(16) are used to compute their counterparts in the sensor frame and, finally, (10)–(12) compute the angles of the new line parametrization in the sensor frame.

The linearised observation matrix is given by:

(32) $$H_{f_{j}}\left(k\right)={\left.\frac{\partial h_{f_{j}}\left(x\left(k\right),v\left(k\right)\right)}{\partial x(k)}\right|}_{x\left(k\right)=\hat{x}\left(k\right)}$$

(33) $$H_{f_{j}}\left(k\right)={\left[\begin{array}{cccccccc} J_{1_{j}}\left(k\right) & 0 & \cdots & 0 & J_{2_{j}}\left(k\right) & 0 & \cdots & 0 \end{array}\right]}_{4\times (9+4n_{l}+3n_{o})}$$

where $J_{1_{j}}$ is a 4×9 Jacobian matrix that represents the partial derivative of transforming $f_{j}$ from the world frame {W} to the sensor frame {S} with respect to the vehicle state, and $J_{2_{j}}$ is a 4×4 Jacobian matrix that represents the partial derivative of transforming $f_{j}$ from the world frame {W} to the sensor frame {S} with respect to the features in the world frame ${}^{W}f_{j}$:

(34) $$J_{1_{j}}\left(k\right)={\left.\frac{\partial h_{j}\left(x_{v}\left(k\right),f_{j}\left(k\right)\right)}{\partial x_{v}\left(k\right)}\right|}_{x_{v}={\hat{x}}_{v}\left(k\right),f_{j}\left(k\right)={\hat{f}}_{j}\left(k\right)}$$

(35) $$J_{2_{j}}\left(k\right)={\left.\frac{\partial h_{j}\left(x_{v}\left(k\right),f_{j}\left(k\right)\right)}{\partial f_{j}\left(k\right)}\right|}_{x_{v}\left(k\right)={\hat{x}}_{v}\left(k\right),f_{j}\left(k\right)={\hat{f}}_{j}\left(k\right)}.$$

Next, observation matrices are stacked to form a single observation matrix:

(36) $$H\left(k\right)={\left[\begin{array}{c} H_{f_{1}}\left(k\right)\\ H_{f_{2}}\left(k\right)\\ \ldots \\ H_{f_{s}}\left(k\right) \end{array}\right]}_{4s\times (9+4n_{l}+3n_{o})}$$

with *s* being the number of observed features. Similarly, the covariance matrices $R_{i}$ are used to form a block diagonal matrix of uncertainty:

(37) $$R\left(k\right)={\left[\begin{array}{cccc} R_{f_{1}}\left(k\right) & 0_{4\times 4} & \ldots & \ldots \\ 0_{4\times 4} & R_{f_{2}}\left(k\right) & \ldots & \ldots \\ \vdots & \vdots & \ddots & 0_{4\times 4}\\ \vdots & \vdots & 0_{4\times 4} & R_{f_{s}}\left(k\right) \end{array}\right]}_{4s\times 4s}.$$

Then, a standard ekf update is applied using these matrices.

#### 4.5.2. New Line Feature Observation

After updating the filter with all the feature observations which have been associated to map features, the remaining non-associated features are considered as candidates to be incorporated to the state vector. Since the structure is known to have only vertical or horizontal pipes, the candidate features are tested against this condition in order to discard outliers.

To add a feature $f_{i}$ observed in the sensor frame {S} to the state vector, it is compounded with the current vehicle position to obtain the feature in the world frame {W}. We denote this operation with the ⊙ operator to distinguish it from the vehicle-point compounding using the ⊕ operator, traditionally defined in the SLAM literature as:

(38) $$f_{j}\left(k\right)=x_{v}\left(k\right)⊙f_{i}\left(k\right)$$

Let the stochastic map at time step *k* be defined by the stochastic vector $x\left(k\right)∼N(\hat{x}\left(k\right),p\left(k\right))$. Then, the augmented state vector, including the new feature, is given by:

(39) $$x_{+}\left(k\right)\equiv N\left({\hat{x}}_{+}\left(k\right),P_{+}\left(k\right)\right)$$

where:

(40) $${\hat{x}}_{+}\left(k\right)={\left[\begin{array}{cc} \hat{x}\left(k\right) & {\hat{x}}_{v}\left(k\right)⊙{\hat{f}}_{i}\left(k\right) \end{array}\right]}^{T}$$

and:

(41) $$p_{+}\left(k\right)=\left[\begin{array}{cc} p(k) & {\left[{p_{v}}^{T}\left(k\right)\;{p_{f_{1}v}}^{T}\left(k\right)\cdots {p_{f_{m}v}}^{T}\left(k\right)\right]}^{T}J_{1⊙}{\left(k\right)}^{T}\\ p_{v}\left(k\right)\;p_{{vf}_{1}}\left(k\right)\cdots p_{{vf}_{m}}\left(k\right)]J_{1⊙}\left(k\right) & J_{1⊙}\left(k\right)p_{v}\left(k\right)J_{1⊙}^{T}\left(k\right)+J_{2⊙}\left(k\right)R_{f_{j}}\left(k\right)J_{2⊙}^{T}\left(k\right) \end{array}\right]$$

where $J_{1⊙}$ is a 4×9 Jacobian matrix that represents the partial derivative of transforming $f_{i}$ from the sensor frame {S} to the world frame {W} with respect to the vehicle state, and $J_{2⊙}$ is a 4×4 Jacobian matrix that represents the partial derivative of transforming $f_{i}$ from the sensor frame {S} to the world frame {W} with respect to the feature in the sensor frame:

(42) $$J_{1⊙}\left(k\right)={\left.\frac{\partial x_{v}\left(k\right)⊙f_{i}\left(k\right)}{\partial x_{v}\left(k\right)}\right|}_{x_{v}={\hat{x}}_{v}\left(k\right),f_{i}\left(k\right)={\hat{f}}_{i}\left(k\right)}$$

(43) $$J_{2⊙}\left(k\right)={\left.\frac{\partial x_{v}\left(k\right)⊙f_{i}\left(k\right)}{\partial f_{i}\left(k\right)}\right|}_{x_{v}={\hat{x}}_{v}\left(k\right),f_{i}\left(k\right)={\hat{f}}_{i}\left(k\right)}$$

Once a feature is added to the state vector, its endpoints are also saved for future re-observations.

### 4.6. Object Feature Observation

From the object semantic segmentation, object features are received as xyz positions in the sensor frame {S}. The first step before the update is the feature association. Already-mapped features in the state vector are transformed to the sensor frame together with their uncertainty. As for the line features, a jcbb algorithm is used to ensure consistency in the associations. Once this association is solved, we have two kinds of observations: re-observed features that were already in the state vector or new features that are candidates to be added to the state vector.

#### 4.6.1. Object Feature Re-Observation

As in the previous case, each feature observation z(k) associated with an already mapped feature $f_{j}$ has an observation Equation (30). In this case, since we use point features instead of lines, a different $h_{j}$ function is used:

(44) $$h_{j}\left(x_{v}\left(k\right),f_{j}\left(k\right)\right)=⊖x_{v}\left(k\right)⊕f_{j}\left(k\right).$$

where ⊕ and ⊖ are the conventional compounding and inverse compounding operations commonly used in the SLAM literature.

Given the point feature observation (32), computing the observation matrix $H_{f_{j}}$ (33) involves computing the Jacobians $J_{1_{j}}$ (34) and $J_{2_{j}}$ (35) of the point feature observation function $h_{j}$ given in (44). In this case, the matrix size is $3\times (9+4n_{l}+3n_{o})$ since the points are tri-dimensional. In a similar way as was used in Section 4.5.1, the stacked observation matrix H(k) can be computed as shown in (36), though, in this case, its dimension is $3s\times (9+4n_{l}+3n_{o})$. Finally, the covariance matrix of the observation can be built as a bloc diagonal matrix as shown in (37) being, in this case, a 3s×3s matrix.

Then, a standard ekf update is applied using these matrices.

#### 4.6.2. New Object Feature Observation

As for the line features, after updating all the object position observations which have been associated to point map features, the remaining non-associated features are considered as candidates to be incorporated to the state vector. The process followed to map the newly discovered objects is equivalent to the one conducted with the pipe lines. The main difference is how the world reference feature position is computed:

(45) $$f_{j}\left(k\right)=x_{v}\left(k\right)⊕f_{i}\left(k\right)$$

which, in this case, uses the conventional vector compounding operation. Therefore, the vector augmentation equations are equivalent to (40) and (41), substituting ⊙ by ⊕, $J_{1⊙}$ by $J_{1⊕}$ and $J_{2⊙}$ by $J_{2⊕}$. Please note that in this case, the $h_{j}$ function used to compute the Jacobians is now the one reported in (44).

## 5. Experimental Results

### 5.1. Experimental Setup

**Figure 9:**
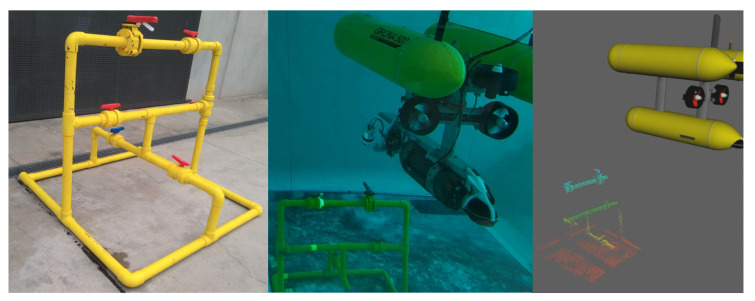
Experimental setup in the water tank with the Girona500 AUV. (**left**) Industrial structure before deployment. (**center**) Underwater view of the water tank during the experiments. (**right**) The 3D visualizer with a scan of the structure.

The underwater test scene consisted of an industrial structure comprising pipes and valves, with an approximate size of 1.4 m width, 1.4 m depth and 1.2 m height (Figure 9). For the testing, this structure was positioned at the bottom of a 5 m deep water tank, while the Girona500 auv [60] moved in a trajectory around it while always facing the underwater structure. The laser scanner measurements were obtained at a distance ranging from 2 to 3.5 m from the underwater structure at a rate of 0.5 Hz. Maintaining a constant distance to the observed structure ensures better results as observed in [61]. The dataset was acquired and stored in a Robot Operating System (ROS) bagfile to be processed offline, consisting of the AUV navigation data (DVL at 5 Hz, pressure at 8 Hz and AHRS at 20 Hz), and the point clouds of 245 laser scans gathered with our laser scanner.

The 245 scans were processed, containing a total of 1268 object observations of 20 unique objects from 6 different classes, and 1778 pipe observations of 12 unique pipes. More details on the experimental setup can be found in [6].

A video showcasing the segmentation and slam results can be found in https://www.youtube.com/watch?v=flFoUrDN-rc (accessed on 27 August 2021).

### 5.2. SLAM Results

**Figure 10:**
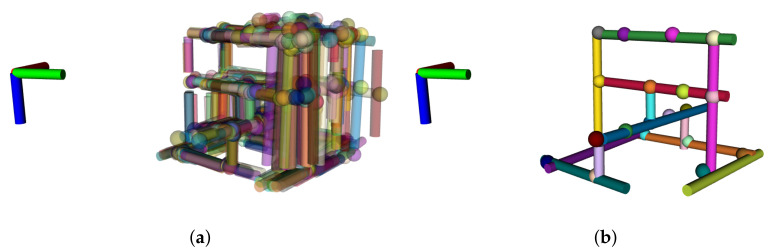
Comparison of maps obtained from DR and SLAM with the NED reference frame. (**a**) DR with 1778 pipes and 1268 objects. (**b**) SLAM with 12 pipes and 20 objects.

The proposed SLAM algorithm with line and object features was compared first with the same algorithm without the feature updates, consisting of a DR navigation. Since no features are used in DR, the resulting map contains all the observations received from the semantic segmentation module (Figure 10a). Nevertheless, the SLAM solution provides a consistent map with all the pipes and objects from the structure (Figure 10b). Note that the lower corner is never observed in this dataset, and thus, the corner object, as well as the full length of the bottom pipes, are not included in the final map.

**Figure 11:**
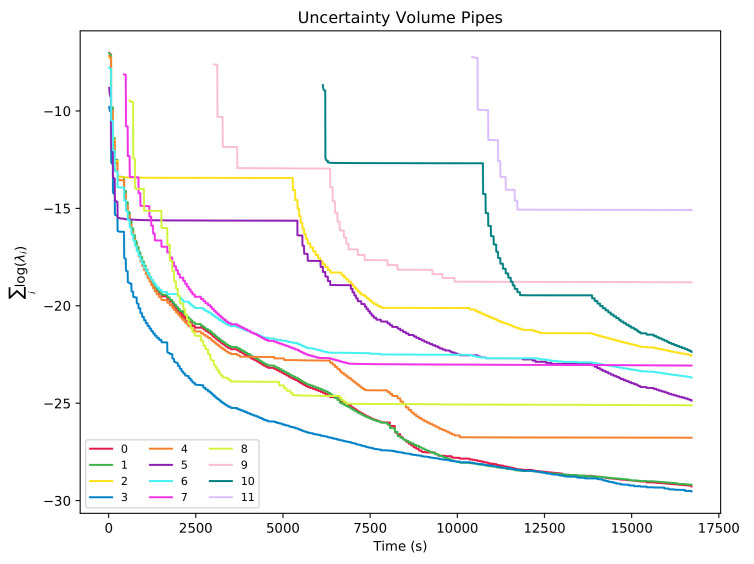
Uncertainty volumes with regards to experiment time for the 12 pipe features.

**Figure 12:**
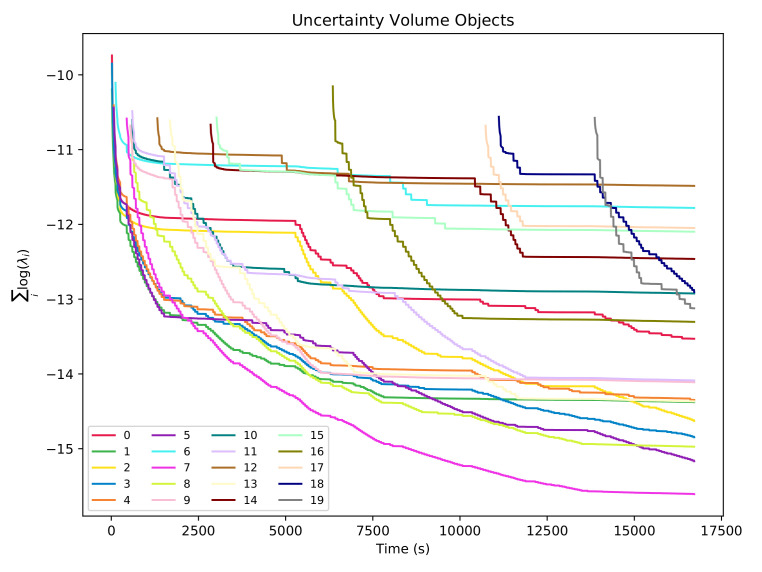
Uncertainty volumes with regards to experiment time for the 20 object features.

To assert the convergence on the state estimation for pipes and objects, one can look at the volume of the uncertainty bounding ellipsoid, which can be computed as $\prod_{i}\lambda_{i}$, where $\lambda_{i}$ are the eigen-values of the uncertainty matrix corresponding to the feature. For better numerical stability, by avoiding multiplications of small numbers that can lead to numerical errors, volumes can be calculated in the logarithmic space as $\sum_{i}\log(\lambda_{i})$. Figure 11 and Figure 12 show how the uncertainty-bounding ellipsoids for each feature decrease through time with each re-observation of the feature and maintain constant values when the features are not re-observed.

**Figure 13:**
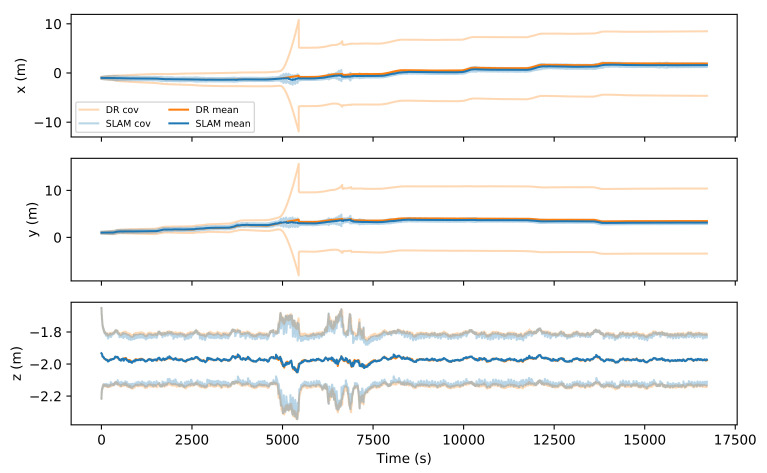
Comparison between DR and SLAM mean values and ±2σ covariances for robot position.

Looking at the vehicle state vector, we can observe that vehicle positions in the xy plane reach a significantly smaller uncertainty than the DR solution (Figure 13).

**Figure 14:**
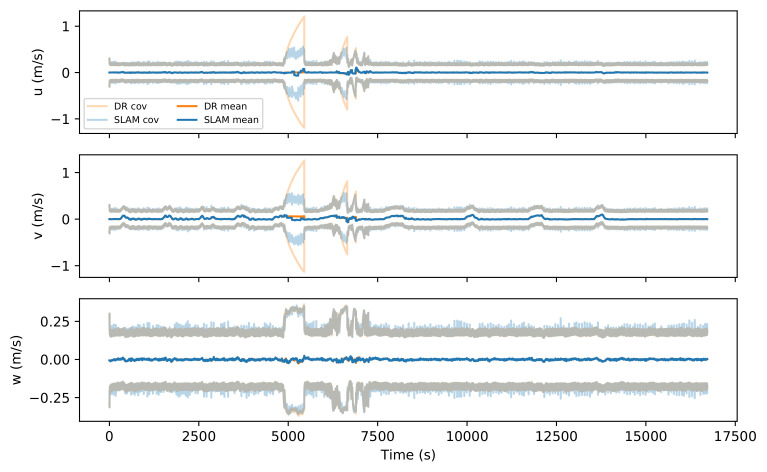
Comparison between dr and SLAM mean values and ±2σ covariances for robot velocity.

It is worth noting that the DR covariance shows several peaks of uncertainty due to the DVL not being able to provide velocity measurements to bound the error. This can be clearly seen in the vehicle state velocity (Figure 14), where the DVL failures are more clearly seen by the growing uncertainty. DVL failures are common in water tank experiments due to the beams impacting the vertical walls. However, the SLAM solution greatly reduces the uncertainty during those events, providing a more accurate estimation.

### 5.3. Object Segmentation Results

**Figure 15:**
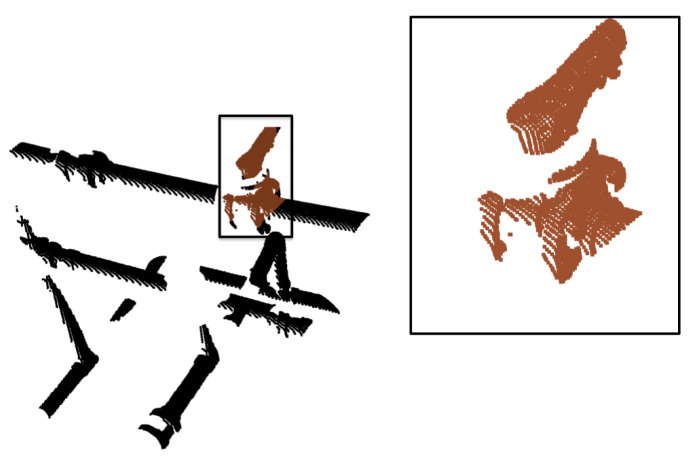
Segmented view of the butterfly valve along with the handle.

Significantly better results have been achieved using the segmentation method described in Section 3.4 compared to our previous solution. The new method correctly segments the handle of the valve, which is a salient feature of this object. This can be appreciated in Figure 15, which is a good example of a *Butterfly* object segmentation. This improvement leads to a better recognition rate with the cvfh descriptor.

### 5.4. Object Recognition Results

**Figure 16:**
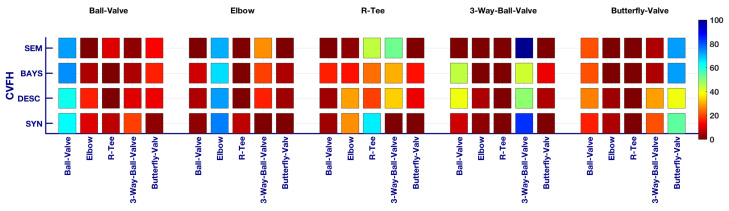
Confusion matrices.

Figure 16 and Table 3 show the confusion matrices of the object recognition method. The row labelled *SYN* shows the confusion matrix, which was computed using synthetic data and the cvfh descriptor only. This confusion matrix is the same as the one presented in [33] and is included here for comparison. The rows labeled *DESC*, *BAYS* and *SEM* show the experimental results of applying the method described in this paper to the dataset reported above. These rows show the confusion matrices when using the cvfh alone, together with the Bayesian estimate, and the result of incorporating the semantic information about the pipe connectivity. The figure shows that, in general, for each row (*SYN*, *DESC*, *BAYS* and *SEM*), the column related to the ground truth class is always the one with the highest recognition rate. It also shows that, in general, the recognition rate grows when incorporating Bayesian estimation and semantic information. Please note that we separate the results (*DESC*, *BAYS* and *SEM*) to provide an insight into how the method works. Nevertheless, the row *SEM*, which corresponds to the output of the complete recognition pipeline, incorporates both Bayesian estimation and semantic information. Therefore, focusing on this row, it can be clearly seen that a good recognition rate is obtained for all objects, with *R-tee* being the most challenging one, since it is often confused with the *3-way-valve*.

On the other hand, Table 4 shows the assessment of the results based on the accuracy, precision, recall and F1 score [62]. Three object classes, namely *Ball-Valve*, *Elbow* and *Butterfly-Valve*, have a balanced trend between recall and precision, resulting in a high F1 score that improves progressively from the descriptor-based to the Bayesian, and then to the semantics-based method.

The *3-Way-Valve* has a high recall, meaning that the system works well recognising it when actually scanning (TP) and that there is a low number of False Negatives (FNs). However, it has a low precision, meaning that the number of FPs is high. Unfortunately, this leads to a poorer F1 score. The high number of FPs (*R-Tees* wrongly detected as *3-way-valves*) may be explained by the fact that most of the *R-Tees* are located at the bottom, on the floor. This means that these objects are far from the laser scanner, and therefore, their point cloud is noisier and of lower resolution (i.e., the point density is considerably lower). The *R-Tees* are particularly sensitive to noise. As can be seen in the database, the object views have smooth continuous curvatures compared to the scanned ones, which produce noisy surfaces. These noisy surfaces distort the results of the descriptor, given that the descriptor is based on the computation of surface normals from the point cloud.

In contrast, the *R-tee* class achieves high precision (low number of FPs) but low recall (high number of FNs) due, again, to the high number of *R-Tees* detected as *3-way-valves*.

## 6. Conclusions

This paper has presented a semantic mapping method using non-coloured point clouds and navigation sensor data. The method includes semantic segmentation (of planes, pipes and objects) paired with a feature-based slam filter and a semantic-based recognition based on multiple views of each tracked object. The methods were tested against real data gathered with an auv in a water tank with a man-made pipe structure.

Semantic segmentation attained better performance in selecting the sets of points belonging to each object than in our previous work. This reduced the negative impact of the presence of points belonging to pipes that made recognition more difficult. The "mushroom" shape bounding box used over the pipe intersections allowed the computation of object candidates with the potential presence of handles, thus enabling a better crop of the input scan that tightly encapsulates the object with handle to be recognized.

Feature-based slam provided an accurate object tracking that allowed the integration of multiple views of the same object acquired at different times in order to better estimate their class. Moreover, it produced a consistent map of the structure while also providing navigation corrections that compensated for the effects of inconsistencies in navigation due to errors in DVL measurements. The integration of the recognition and the slam module, where information is passed back and forth, was instrumental to the higher performance of the approach and to the ability to create a semantic map of all recognized objects.

## 7. Future Work

Future research plans will continue in the direction of combining the representations of slam and object recognition to provide a more accurate and detailed 3D semantic map while providing recognition with more complete views. The object recognition approach we used, based on SLAM, mainly consists of two modules: a Bayesian semantic information-based method for recognition and the SLAM system. Since SLAM provides long-term consistent navigation, one future improvement will be to use this navigation to fuse several scans, which will provide more comprehensive views of the objects. Having more complete views has the potential to improve both the accuracy of object recognition and the reliability of pose estimates, especially in challenging scenarios with significant changes in viewpoints.

A longer term strategy to improve the observation quality is to perform view planning in order to reduce the ambiguity caused by poorly observed objects. Such view planning should be multi-objective in the sense of taking into account multiple objects simultaneously and should be guided towards the next best views that solve the ambiguity between the most probable classes for each object. Continuing this work, future efforts will be directed toward the goal of grasping and manipulating such objects.

## Figures and Tables

**Table 1:** Polyvinylchloride (PVC) pressure pipe objects used in the experiments (reprinted with permission from ref. [6] 2021 Sensors).

PVC Objects	Id Name	Size (mm${}^{3}$)	PVC Objects Views (12)
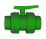	1-Ball-Valve	198×160×120	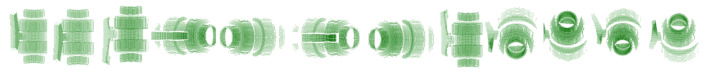
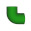	2- Elbow	122.5×122.5×77	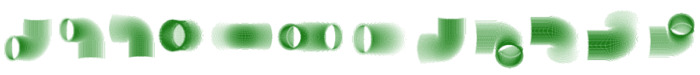
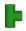	3- R-Tee	122.5×168×77	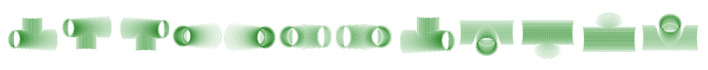
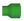	4- R-Socket	88×75×75	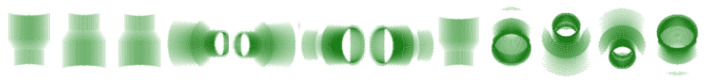
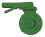	5- Butterfly-Valve	287.5×243×121	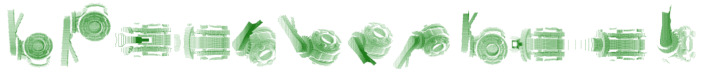
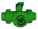	6- 3-Way-Ball-Valve	240×160×172	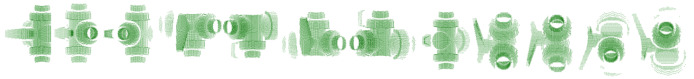

**Table 2:** Semantic connection of objects. The number of pipes connected to an object is indicated by $n_{p}$ (reprinted with permission from ref. [6]. 2021 Sensors).

Type of Connection	Pipe Disposition			Potential Object Candidates
	$n_{p}$	‖	*⊥*	
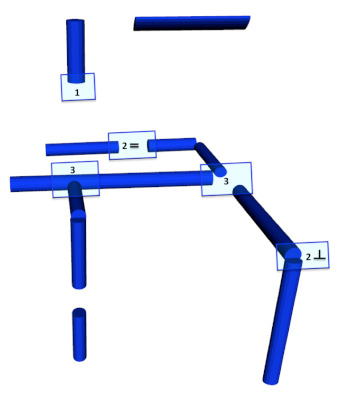	3	2	1	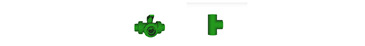
	2	0	2	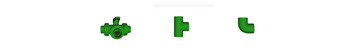
	2	2	0	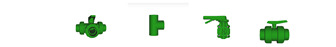
	1|0	1|0	1|0	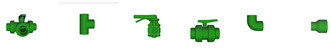

**Table 3:** Confusion matrices expressed as a numerical %. The objects 1, 2, 3, 4, 5 represent, respectively: a *Ball-Valve*, an *Elbow*, a *R-Tee*, a *3-Way-Valve*, and a *Butterfly-Valve*. We highlighted the best recognition for each object, which coincides with the correct class and the usage of semantic information.

Descriptors	Experiment	Objects																								
		Ball Valve					Elbow					R-Tee					3-Way-Ball-Valve					Butterfly-Valve				
		1	2	3	4	5	1	2	3	4	5	1	2	3	4	5	1	2	3	4	5	1	2	3	4	5
**CVFH**	**SYN**	63	10	7	19	2	2	75	7	1	1	4	27	65	1	1	9	3	1	84	1	17	5	1	21	54
	**DESC**	60.46	18.11	1.28	9.44	10.71	2.74	80.82	4.11	9.59	2.74	1.68	38.13	19.18	28,78	12.23	39.64	5.41	0.9	49.55	4.5	21.76	12.35	7.06	25.29	33.53
	**BAYS**	82.65	0	0	0	17.35	1.37	90.41	1.37	6.85	0	6.47	10.55	51.32	19.42	12.23	1.8	0	0	95.5	2.7	0	0	0	0	100
	**SEM**	82.65	0	0	0	17.35	0	73.97	17.81	8.22	0	0	3,12	64.75	32,13	0	0	0	0	100	0	0	0	0	0	100

**Table 4:** Assessment of the recognition performance through accuracy, recall, precision and F1 score. We highlighted the best results in blue color, which are all consistent with the use of semantic information, except for *3-Way-Ball-Valve*, where the best F1 score was achieved using the Bayesian estimation method.

Descriptors	Experiment	Objects																							
		Average				Ball Valve				Elbow				R-Tee				3-Way-Ball-Valve				Butterfly-Valve			
		Accuracy	Recall	Precision	F1-Score	Accuracy	Recall	Precision	F1-Score	Accuracy	Recall	Precision	F1-Score	Accuracy	Recall	Precision	F1-Score	Accuracy	Recall	Precision	F1-Score	Accuracy	Recall	Precision	F1-Score
**CVFH**	**DESC**	0.42	0.49	0.46	0.38	0.4	0.60	0.72	0.66	0.42	0.81	0.19	0.30	0.42	0.19	0.79	0.31	0.42	0.50	0.21	0.29	0.42	0.34	0.36	0.35
	**BAYS**	0.76	0.84	0.73	0.74	0.76	0.83	0.92	0.87	0.76	0.90	0.60	0.72	0.76	0.51	1	0.68	0.76	0.95	0.55	0.70	0.76	1	0.58	0.74
	**SEM**	0.80	0.84	0.78	0.78	0.8	0.83	1	0.91	0.80	0.74	0.81	0.77	0.80	0.65	0.95	0.77	0.80	1	0.44	0.61	0.80	1	0.71	0.83

## Data Availability

The data presented in this study are available on request from the corresponding author.
